# Natural compounds isolated from Brazilian plants are potent inhibitors of hepatitis C virus replication in vitro

**DOI:** 10.1016/j.antiviral.2014.12.018

**Published:** 2015-03

**Authors:** A.C.G. Jardim, Z. Igloi, J.F. Shimizu, V.A.F.F.M. Santos, L.G. Felippe, B.F. Mazzeu, Y. Amako, M. Furlan, M. Harris, P. Rahal

**Affiliations:** a UFU – Federal University of Uberlândia, Institute of Biomedical Science – ICBIM, Uberlândia, MG, Brazil; b UNESP – São Paulo State University, Institute of Bioscience, Language and Exact Science – IBILCE, Department of Biology, São José do Rio Preto, SP, Brazil; c School of Molecular and Cellular Biology, Faculty of Biological Sciences and Astbury Centre for Structural Molecular Biology, University of Leeds, Leeds LS2 9JT, United Kingdom; d UNESP – São Paulo State University, Institute of Chemistry, Department of Organic Chemistry, Araraquara, SP, Brazil; e Department of Microbiology and Cell Biology, Tokyo Metropolitan Institute of Medical Science, Tokyo, Japan

**Keywords:** Brazilian plants, Natural compounds, Antiviral, Hepatitis C virus, Replication

## Abstract

Compounds extracted from plants can provide an alternative approach to new therapies. They present characteristics such as high chemical diversity, lower cost of production and milder or inexistent side effects compared with conventional treatment. The Brazilian flora represents a vast, largely untapped, resource of potential antiviral compounds. In this study, we investigate the antiviral effects of a panel of natural compounds isolated from Brazilian plants species on hepatitis C virus (HCV) genome replication. To do this we used firefly luciferase-based HCV sub-genomic replicons of genotypes 2a (JFH-1), 1b and 3a and the compounds were assessed for their effects on both HCV replication and cellular toxicity. Initial screening of compounds was performed using the maximum non-toxic concentration and 4 compounds that exhibited a useful therapeutic index (favourable ratio of cytotoxicity to antiviral potency) were selected for extra analysis. The compounds APS (EC_50_ = 2.3 μM), a natural alkaloid isolated from *Maytrenus ilicifolia*, and the lignans 3^∗^43 (EC_50_ = 4.0 μM), 3^∗^20 (EC_50_ = 8.2 μM) and 5^∗^362 (EC_50_ = 38.9 μM) from *Peperomia blanda* dramatically inhibited HCV replication as judged by reductions in luciferase activity and HCV protein expression in both the subgenomic and infectious systems. We further show that these compounds are active against a daclatasvir resistance mutant subgenomic replicon. Consistent with inhibition of genome replication, production of infectious JFH-1 virus was significantly reduced by all 4 compounds. These data are the first description of Brazilian natural compounds possessing anti-HCV activity and further analyses are being performed in order to investigate the mode of action of those compounds.

## Highlights

• Antiviral effects of 20 Brazilian natural compounds were investigated.
• Four compounds with potent inhibitory activity on HCV replication were identified.
• Antiviral activity was independent of HCV genotype.
• Antiviral effect was not affected by variants described to confer resistance.
• One of the four compounds inhibited HCV IRES-mediated translation.

## 1 Introduction

Hepatitis C virus (HCV) infection is a worldwide public health problem and it is estimated that the virus infects around 3% of the world population (Shepard et al., 2005). Chronic infection can progress to liver cirrhosis with risk of the development of hepatocellular carcinoma, and causes around 500,000 deaths per year (Alter, 2007; Chevaliez and Pawlotsky, 2007; Saito et al., 1990). There is no effective vaccine for prevention of HCV infection; however a number of drugs are available for the treatment of infection. Until recently, the standard therapy was based on pegylated interferon (IFN) plus ribavirin (RBV), resulting in a sustained virological response in approximately 50% of patients infected with HCV genotypes 1a/1b and 80% of those infected with genotypes 2 or 3 (Fried et al., 2002; Hadziyannis et al., 2004; Manns et al., 2001). The availability of new, direct-acting antivirals targeting the NS3 protease, NS5B polymerase and NS5A protein have dramatically improved therapeutic options (Pawlotsky, 2014). However, the high costs and potential for development of resistance presented by existing treatment demonstrate the need for the development of more efficient new antivirals, or combination of therapies for HCV treatment.

Traditional medicines have a long history and there is now a great interest in discovering new molecules from natural sources for the treatment of many human diseases. An extensive variety of natural compounds has demonstrated antiviral action worldwide, including anti-HCV activity (Calland et al., 2012). In this context, compounds extracted from plants can provide an alternative approach to new therapies. Natural compounds present characteristics such as high chemical diversity, lower cost of production and milder or non-existent side effects than conventional treatment (Kitazato et al., 2007). Additionally, most of the drugs used today in the clinic were first discovered from plants and microorganisms (Mann, 2002). Therefore, they present a great opportunity to find novel compounds that can act as antiviral drugs.

The Brazilian flora represents a vast, largely untapped, resource of potential therapeutic compounds. The wide distribution of natural resources in Brazil and the natural diversity of chemical components provide the country with potential bioactive materials (Duarte et al., 2005). Here we investigate the antiviral effects of a panel of Brazilian natural compounds consisting of extracts, fractions and isolated compounds on HCV replication. These data are the first description of Brazilian natural compounds possessing anti-HCV activity.

## 2 Materials and methods

### 2.1 Natural compounds

Compounds were extracted from *Maytrenus ilicifolia* (APS, C, P and M), *Peperomia blanda* (5-362, 3-20, 3-43, 48-3, F3 and F6) and *Piper fuligineum* (F8–40). The root bark of *M. ilicifolia* was collected in the city of Ribeirão Preto (São Paulo State, Brazil, at 21°11′56.1″S; 47°46′42.2″W) in March 2006. The plant was identified by Rita Maria de Carvalho. A voucher specimen (HPM-BR 0059) has been deposited in the Herbarium of the University of Campinas, São Paulo, Brazil (Santos et al., 2012). The aerial parts of *P. blanda* were collected at the Reserva da Ripasa, Ibaté – SP, Brazil in January of 2005 and identified by Dr. Elsie Franklin Guimarães. A voucher specimen (Kato-547) has been deposited at the Herbarium of the Institute of Bioscience, São Paulo University, São Paulo – SP, Brazil (Felippe et al., 2008). The *Piper fuligineum* species was identified by Dr. Agnes Lamb of the Institute of Botany (IBt of São Paulo, SP, Brazil) and their voucher specimens are deposited in the Herbarium of the Institute of Botany (USP – SP) under the voucher Kato-0720.

**Fig. 1:**
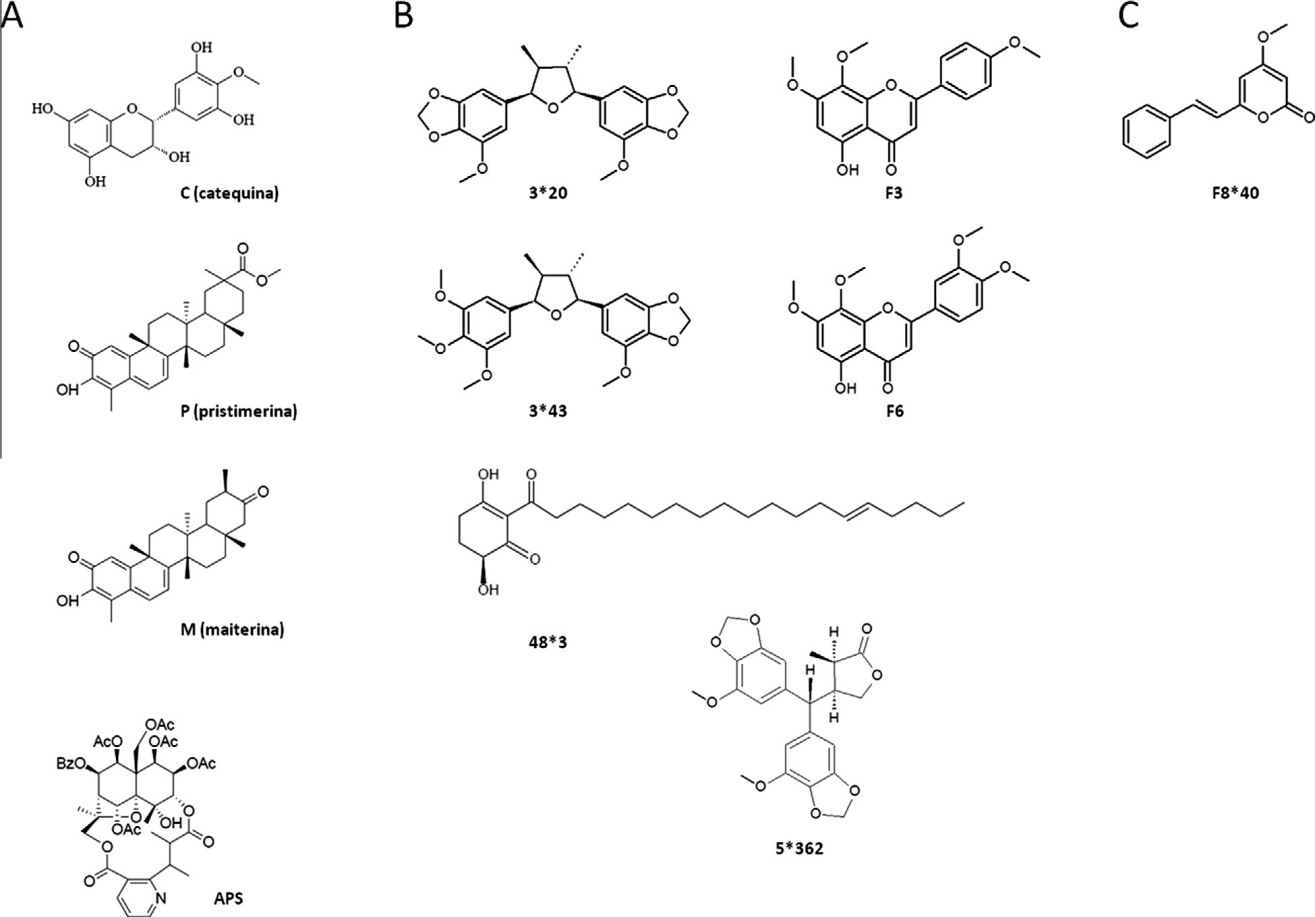
Chemical structure of Brazilian natural compounds. Compounds isolated from *Maytrenus ilicifolia* (A), *Peperomia blanda* (B) and *Piper fuligineum* (C).

The full details of compound extraction and purification was described previously (Costa et al., 2008; Dos Santos et al., 2013; Felippe et al., 2008, 2012; Gullo et al., 2012; Santos et al., 2012) and the structures of isolated compounds are shown in Fig. 1. The compounds were dissolved in dimethyl sulfoxide (DMSO, Sigma–Aldrich) as stock solutions stored at −20 °C. Dilutions of the compounds in complete medium were made immediately prior to the experiments to reach a maximum final concentration of 0.5% DMSO. For all the assays performed, control cells were treated with medium added with DMSO at the final concentration of 0.5%. Cyclosporin A (CsA, Sigma–Aldrich) was used as a positive control for inhibition of replication.

### 2.2 Cell culture

Huh7.5 cells were cultured in Dulbecco’s modified Eagle’s medium (DMEM; Sigma–Aldrich) supplemented with 10% fetal calf serum, 100 IU penicillin ml^−1^, 100 μg streptomycin ml^−1^ and 1% non-essential amino acids at 37 °C in a humidified 5% CO_2_ incubator. Subgenomic replicon (SGR) harboring cell lines (genotype 2a SGR-Feo-JFH-1 (Wyles et al., 2009), genotype 1b SGR-Feo-BM4-5 (Wyles et al., 2007) and (genotype 3a – Genbank GU814264 (Saeed et al., 2012)) were maintained in DMEM with 300 μg/mL G418.

### 2.3 Cytotoxicity assay

Cell viability was measured by the MTT [3-(4,5-dimethylthiazol-2-yl)-2,5-diphenyl tetrazolium bromide] (Sigma–Aldrich) method. Huh7.5 cells or SGR-harboring cell lines were cultured in DMEM medium in a 96-multi-well plate and incubated at 37 °C in a humidified 5% CO_2_ incubator overnight. Drug-containing medium at different concentrations was added to the cell culture being replaced every 24 h. After 48 h incubation at 37 °C, DMEM containing MTT at the final concentration of 1 mg/mL was added to each well, incubated for 1 h and replaced with 100 μl of DMSO to solubilize the formazan crystals. Surviving cells were measured by optical density (OD) of each well at 570 nm, using a spectrophotometer. Cells viability was calculated according to the equation (*T*/*C*) × 100%, where *T* and *C* represent the mean optical density of the treated group and control group, respectively. All experiments were performed in triplicates and repeated at least three times. Further assays were performed considering 80% viability of treated cells.

### 2.4 Luciferase-based replication assay

T7 transcripts were generated from linearized DNA templates of SGR-luc-JFH-1, SGR-luc JFH-1 containing the NS5A Y93H Daclatasvir (DCV) resistance mutation or SGR-luc-JFH-1/GND luciferase subgenomic replicons (SGR) (Targett-Adams and McLauchlan, 2005). 4 × 10^6^ Huh7.5 cells were washed and resuspended in diethylpyrocarbonate (DEPC)-treated PBS, and electroporated with SGR RNA (2–5 μg) in 0.4 cm cuvettes at 950 μF, 270 V. Cells were seeded into 96-well plates at a density of 8 × 10^3^ per well and compounds were added at 2–4 h post-electroporation. Cells were harvested by lysis with Passive Lysis Buffer (Promega) at 4, 16, 24 and 48 h post-electroporation and HCV RNA replication was quantified by measuring luciferase activity using the Luciferase Assay System (Promega). The same assays were performed with SGR-harboring cell lines (genotype 2a SGR-Feo-JFH-1) for comparison. The effective concentration 50% (EC_50_) was calculated using Prism (GraphPad) and cytotoxicity assays were carried out in parallel to determine the cytotoxic concentration 50% (CC_50_) using a MTT-based system as described below. The values of CC_50_ and EC_50_ were used to calculate the selectivity index (SI = CC_50_/EC_50_), which suggests the potential antiviral activity of the compounds. SI with value of four or higher suggests that a compound have a promising antiviral activity that merit further studies.

Huh7.5 cells stably harboring the SGR-Feo-BM4-5 (Wyles et al., 2007) (genotype 1b) or SGR-Feo-S52 (genotype 3a) culture adapted mutants AII (T1056A, T1429I and S2204I) or SHI (P1220S, D1430H and S2204I) (Saeed et al., 2012) were seeded in a 96 well plates at the same cell density. Cells were treated at 4 h post seeding for 48 h with the previously determined concentration of compounds or DCV, harvested and luciferase measured.

### 2.5 Virus assays

For virus replication assays, 8 × 10^6^ Huh7.5 cells were electroporated with 10 μg of Rluc-J6/JFH1 (mFL-J6/JFH-5′C19Rluc2AUbi) (Tscherne et al., 2006). Compounds were added at 2–4 h post-electroporation. Samples were harvested in Renilla lysis buffer (Promega) at 48 h post-electroporation and virus replication was quantified by measuring luciferase activity using the Renilla Luciferase Assay System (Promega).

For infection assays, Huh7.5 cells were seeded the day before the assay was carried out. Compounds were diluted to the stated final concentrations in DMEM media. Two types of experiments were carried out; Cells were infected with Rluc-J6/JFH1 virus and compounds were added. After 48 h samples were harvested and luminescence was measured. Alternatively, cells were infected with JFH1 virus (Wakita et al., 2005) for 4 h, washed extensively to remove virus and treated with the compounds. After 48 h extra cellular virus was titrated. The titer plate was fixed with 4% PFA after 48 hpi and stained for NS5A using sheep anti-NS5A (Macdonald et al., 2003) and Alexa Fluor anti-sheep 594 secondary antibody.

### 2.6 Western blot analysis

Cells were lysed in Glasgow lysis buffer [GLB; 10 mM Pipes–KOH (pH7.2), 120 mM KCl, 30 mM NaCl, 5 mM MgCl^2^, 1% Triton X-100 (Sigma), 10% glycerol] (Ross-Thriepland and Harris, 2014) plus protease and phosphatase inhibitors (2 mM Na_3_VO_4_, 5 mM NaF, 5 mM Na_4_P_2_O_7_). Fifty micrograms of protein were resolved by SDS/PAGE and transferred to a PVDF membrane using a semidry transfer apparatus. Membranes were blocked in 10% (w/v) dried skimmed milk powder in Tris-buffered saline with 0.1% Tween-20 (TBS-T). Membranes were probed with anti-NS5A (Macdonald et al., 2003) or mouse anti-GAPDH (AbCam) in 5% (w/v) dried skimmed milk in TBS-T. The antibodies were detected with the relevant secondary horseradish peroxidase-conjugated antibody and in-house enhanced chemiluminescent reagent.

### 2.7 Statistical analysis

Individual experiments were performed in triplicate and all assays were performed a minimum of three times in order to confirm the reproducibility of the results. Differences between means of readings were compared using analysis of variance (one-way or two-way ANOVA) and Student *t* test. *P* values of less than 0.05 (indicated by asterisks) were considered to be statistically significant.

## 3 Results

### 3.1 Screening of compounds isolated from Brazilian plants for effects on HCV replication

**Supplementary Fig. S1:**
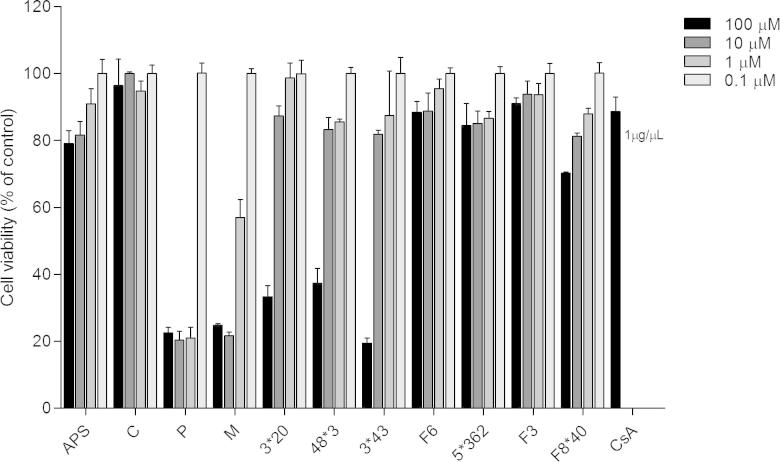
Huh7.5 cells were treated with 100, 10, 1 or 0.1 μM of each compound and incubated for 48 h to access the cytotoxicity of the compounds. Cell viability was measured by MTT assay. Values were normalized to the non-treated control. CsA (1 μg/ml) was used as a positive control. Mean values of three independent experiments each measured in triplicate including the standard deviation are shown.

**Fig. 2:**
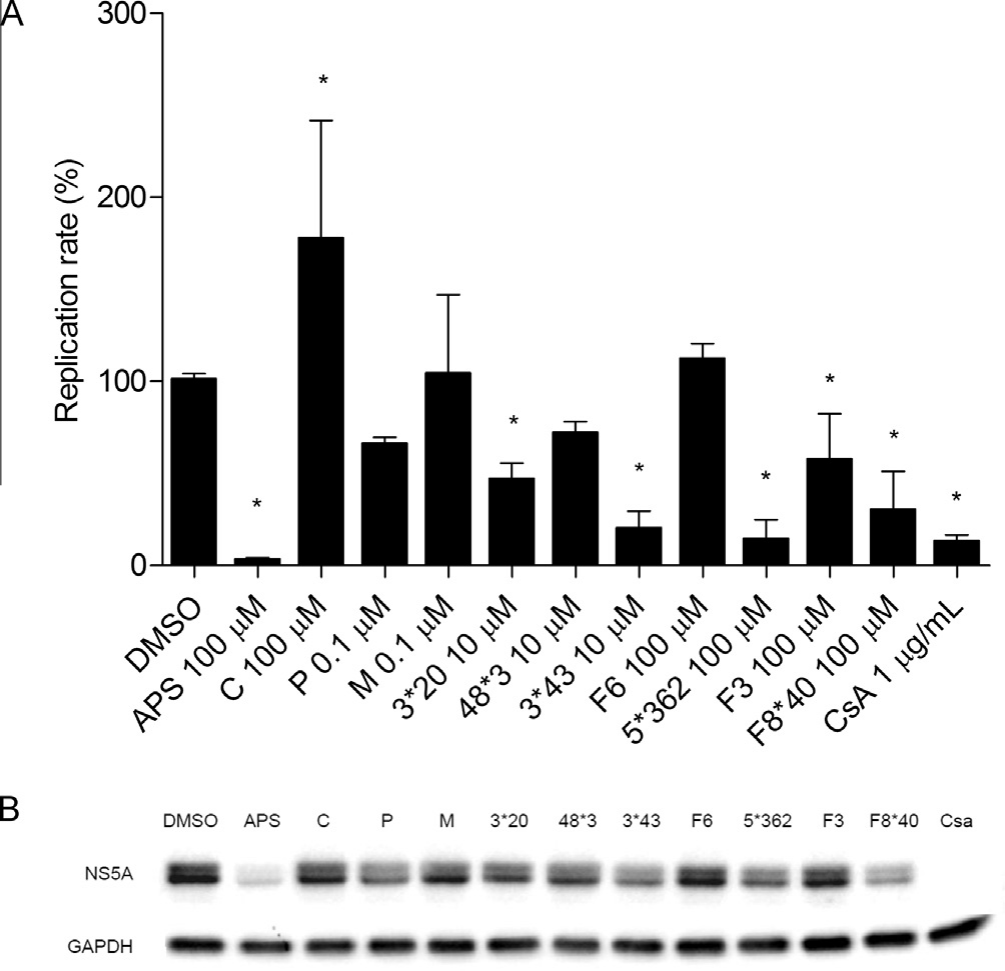
Screening of plant-derived compounds for activity against HCV replication. Huh7.5 cells were electroporated with SGR-luc-JFH1, and 4 h later, specific concentrations of compounds were added. Replication efficiency was measured 48 h post-electroporation using luciferase (A) and western blotting assays (B). DMSO and cyclosporine A were used as negative and positive controls respectively. Mean values of three independent experiments each measured in triplicate including the standard deviation are shown. *P* < 0.05 was considered significant.

To evaluate whether a panel of Brazilian natural compounds (Fig. 1) could inhibit HCV replication, we performed a screening assay using a firefly luciferase SGR construct (SGR-luc-JFH1). Initially, Huh7.5 cells were treated with 100, 10, 1 or 0.1 μM of each compound and incubated for 48 h to assess the cytotoxicity of the compounds (Fig. S1). Then, Huh7.5 cells were electroporated with SGR-luc-JFH1 and compounds were added to the cells at 4 h post-electroporation. Replication levels were assessed 48 h later by luciferase assay. The initial data showed that the purified compounds APS, 3^∗^43, 3^∗^20, 5^∗^362, F3 and F8^∗^40 (Fig. 1) significantly inhibited HCV SGR replication (*p* < 0.05) (Fig. 2A). Expression of NS5A was also significantly reduced in the presence of APS, 3^∗^43, 3^∗^20, 5^∗^362, F3 and F8^∗^40 as shown in Fig. 2B. This analysis also revealed that the compounds had no significant effect on the phosphorylation profile of NS5A, as both basal and hyper phosphorylated forms could be seen. Intriguingly, treatment of cells with compound C appeared to significantly enhance replication (*p* < 0.05) with a concomitant increase in protein expression (Fig. 2B). These results demonstrated that most of the selected Brazilian natural compounds are able to inhibit HCV replication.

### 3.2 Inhibitory effect of Brazilian natural compounds on HCV replication

**Supplementary Fig. S2:**
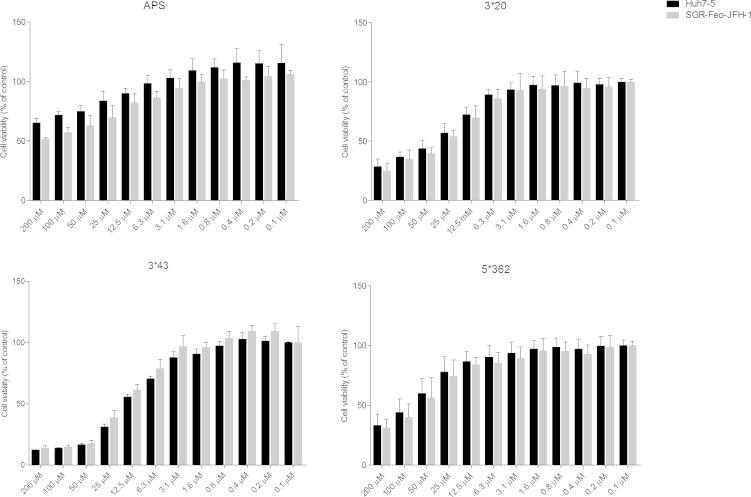
Huh7.5 cells or Huh7.5 cell line stably harboring the SGR-Feo-JFH-1 replicon was treated with increasing concentration of compounds (0.1–200 μM) and cell viability was measured by MTT assay at 48 h after compound addition. Values were normalized to the non-treated control. Mean values of three independent experiments each measured in triplicate including the standard deviation are shown.

**Fig. 3:**
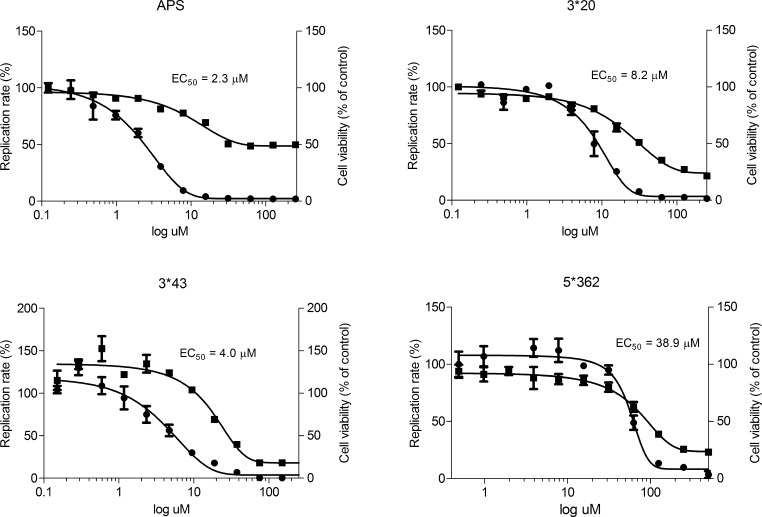
Determination of EC_50_ for compounds APS, 3^∗^20, 3^∗^43 and 5^∗^362. Huh7.5 stably harboring SGR-Feo-JFH-1 were incubated with compounds at concentrations over a 3-log range for 48 h. Replication efficiency was measured by luciferase assay (indicated by ●) and cellular viability measured using an MTT assay (indicated by ■). Mean values of three independent experiments each measured in triplicate including the standard deviation are represented.

For further analysis we selected four compounds, APS, 3^∗^43, 3^∗^20 and 5^∗^362, as these showed significant inhibition of HCV genome replication at non-cytotoxic concentrations. An Huh7.5 cell line stably harboring the SGR-Feo-JFH-1 replicon was treated with increasing doses of compounds and replication efficiency and cell viability were measured 48 h after compound addition. The results indicated that all four compounds APS, 3^∗^43, 3^∗^20 and 5^∗^362 decreased HCV replication in a dose-dependent manner with EC_50_ of 2.3, 4.0, 8.2 and 38.9 μM, respectively (Table 1; Figs. 3 and S2). We also assayed the compounds F3 and F8^∗^40 however we were not able to establish EC_50_ for those compounds. They reduced replication only when cytotoxic concentrations were used (data not shown) and were therefore excluded from further analysis. Subsequent studies focused on compounds APS, 3^∗^43, 3^∗^20 and 5^∗^362.

### 3.3 Effect of the compounds on HCV IRES driven translation

**Fig. 4:**
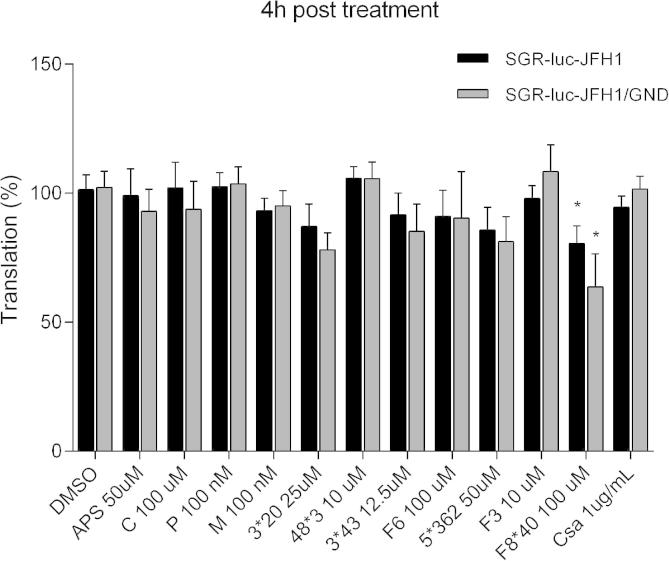
Effects of compounds APS, 3^∗^20, 3^∗^43 and 5^∗^362 on HCV IRES-mediated translation. Huh7.5 cells were transfected with in vitro transcripts of the SGR-luc-JFH1 or the SGR-luc-JFH-1 (GND) and compounds were added immediately. Translation levels of WT and mutant constructs are shown at 4 h post-electroporation. Mean values of three independent experiments each measured in triplicate including the standard deviation are shown. *P* < 0.05 was considered significant.

We next assessed the impact of natural compounds on HCV-RNA translation, also considering compounds which did not present effects on replication in the previous assays. To this end, we transfected Huh7.5 cells with in vitro transcribed RNA of SGR-luc-JFH1 or the SGR-luc-JFH1 (GND) polymerase-defective construct (containing a mutation of the conserved GDD motif to GND) and compounds were added immediately. Luciferase values of both WT and GND constructs are shown at 4 h, which was representative of input translation. The results demonstrated that the treatment with most of the compounds did not affect HCV IRES driven translation (Fig. 4). As an exception, the compound F8^∗^40 showed a modest yet significant reduction of luciferase levels to 80.6% (*p* < 0.05), suggesting that this compound can have a slight effect on IRES-directed translation. These data corroborate with a reduction in protein levels observed in the presence of F8^∗^40 (Fig. 2B).

### 3.4 Compounds APS, 3^∗^43, 3^∗^20 and 5^∗^362 prevent replication complex formation

**Fig. 5:**
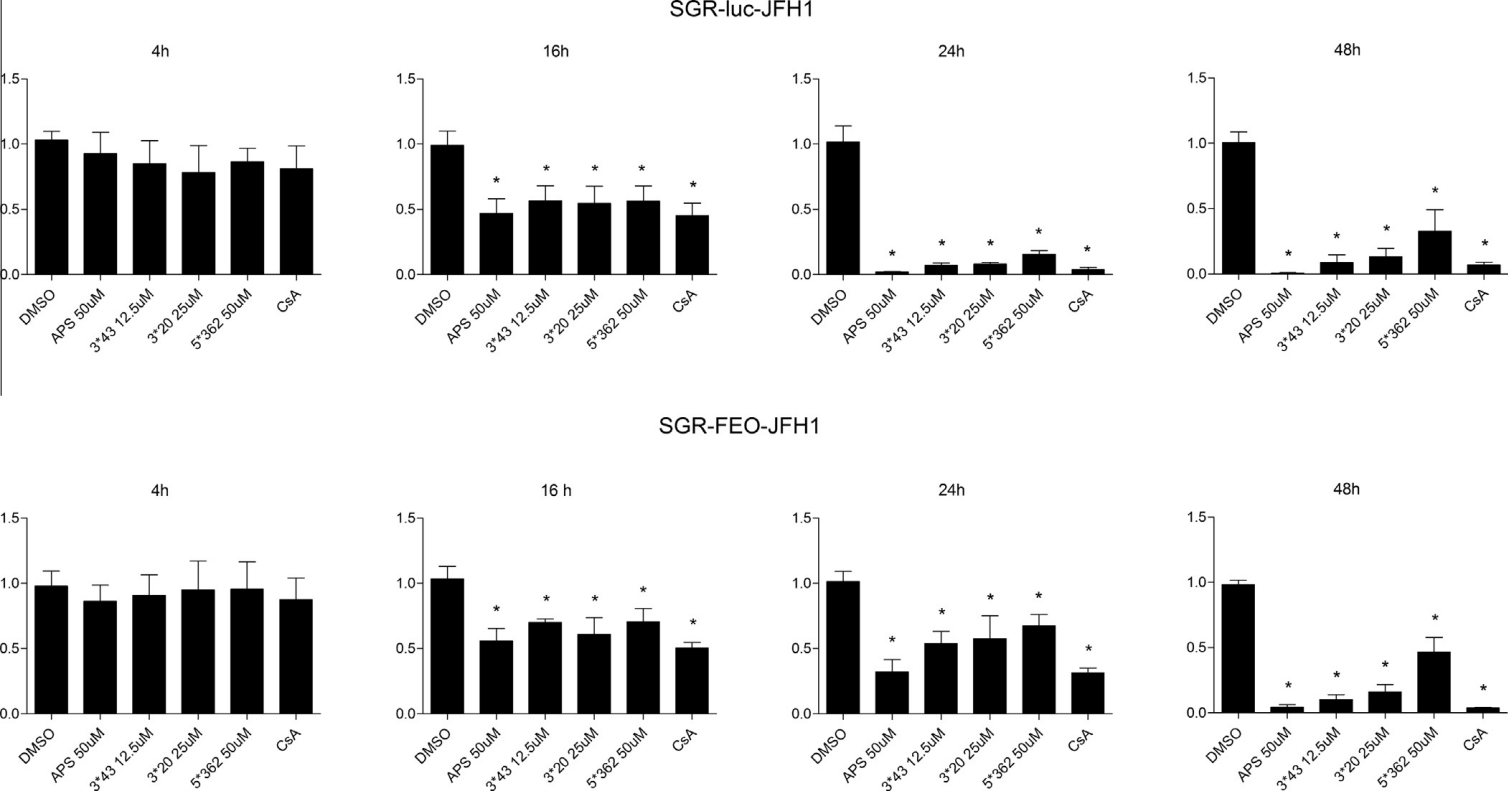
Effects of compounds APS, 3^∗^20, 3^∗^43 and 5^∗^362 on formation of HCV genome replication complexes. Huh7.5 cells were electroporated with SGR-luc-JFH1, and 2 h later, specific concentrations of compounds were added. Replication efficiency was measured at 4, 16, 24 and 48 h post-electroporation using luciferase to assess the effect of compounds in preventing the formation of RCs (upper panel). Huh7.5 harboring SGR-Feo-JFH-1 were treated with indicated concentrations of compounds for 48 h. Replication efficiency was assessed to check the effect of compound on pre-existing RCs (lower panel). Mean values of three independent experiments each measured in triplicate including the standard deviation are shown. *P* < 0.01 was considered significant.

We wished to investigate whether compounds APS, 3^∗^43, 3^∗^20 and 5^∗^362 acted either on pre-existing replication complexes (RC), or by inhibiting their formation. Huh7.5 cells were electroporated with SGR-luc-JFH-1 RNA and compounds were added to the cells at 2 h post-electroporation at the defined concentrations. RNA replication was monitored for 48 h by luciferase assay in order to detect the ability of compounds to prevent RC formation. In parallel, Huh7.5 cells stably expressing SGR-Feo-JFH-1 replicons were treated with compounds and harvested at the same time points to evaluate the activity on pre-existing RCs. No significant reduction of replication levels was observed in either assay at 4 h. For both transient and stable replicons, replication decreased significantly compared to DMSO control from 16 h post-electroporation but there was no difference between the two assays (Fig. 5). In contrast, at 24 h there was marked difference between the levels of inhibition observed in the transient and stable assay formats. Specifically, the compounds were more effective on the transient replicons. At a later time point (48 h) again no difference was observed. These data are consistent with the hypothesis that these compounds block formation of RCs and have a lesser effect on pre-existing RCs. In the transient assay the luciferase levels at 4 h reflect translation from input RNA whereas luciferase activity at 24 h is a measure of RNA produced by newly formed RCs. After this time point, replication was gradually reduced over time up to 48 h, showing that the compounds were preventing replication.

The luciferase levels detected at 4 h in the stable replicon cells reflects replication by pre-existing active RCs and did not respond to treatment with the compounds. Values at 16 and 24 h reflect both pre-existing and newly formed RCs and are not affected as efficiently as the corresponding values in the transient assay, consistent with the hypothesis that the compounds are predominantly inhibiting RC formation. However, we acknowledge that interpretation of these experiments is challenging and we therefore cannot rule out the possibility that these compounds inhibit both RC formation and activity.

### 3.5 Activity of compounds APS, 3^∗^43, 3^∗^20 and 5^∗^362 against a DCV resistant JFH-1 SGR and genotypes 1b/3a SGRs

**Fig. 6:**
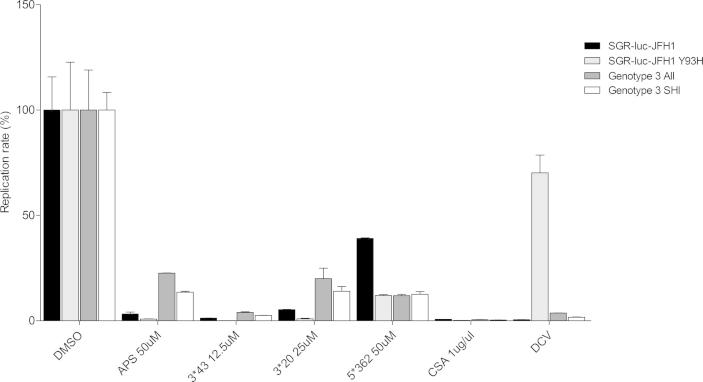
Activity of compounds APS, 3^∗^43, 3^∗^20 and 5^∗^362 against a DCV resistant mutant and genotypes 1b/3a SGRs. Huh7.5 cells electroporated with SGR-luc-JFH-1 or a corresponding NS5A Y93H DCV resistance mutation containing RNA, or Huh7.5 cells stably harboring the SGR-Feo genotype 3a AII (T1056A, T1429I and S2204I) or SHI (P1220S, D1430H and S2204I) (Saeed et al., 2012) culture adaptive mutations were treated 4 h post electroporation/seeding for 48 h with the previously determined concentration of compounds or of DCV (JFH1: 17.6 pM; genotype 3a AII: 5.2 nm and SHI 2.4 nm), harvested and luciferase measured. *P* < 0.05 was considered significant.

Next, we investigated whether compounds APS, 3^∗^43, 3^∗^20 and 5^∗^362 were able to block replication of an SGR that was resistant to one of the DAAs in current use. The most potent of these is daclatasvir (DCV) with an EC_50_ against HCV replication of less than 100 ρM (Gao et al., 2010) – however a single point mutation (Y93H in NS5A) results in ∼1000-fold loss of sensitivity to DCV. Huh7.5 cells were therefore electroporated with SGR-luc-JFH1 WT or Y93H RNA and seeded in a 96 well plate. Cells were incubated from 4 to 48 h post seeding in the presence of either APS, 3^∗^43, 3^∗^20, 5^∗^362 or DCV (17.6 pM), prior to lysis and measurement of luciferase activity. Reassuringly, all 4 compounds significantly inhibited both WT and Y93H SGR replicon to similar levels (*p* < 0.05) (Fig. 6).

We also evaluated the ability of the compounds to inhibit the replication of alternative genotypes of HCV. To do this we chose genotype 3a as this is increasingly common and is inherently more resistant to the new DAAs. As transient SGR for genotype 3a are not available, we utilized Huh7.5 cells stably harboring the genotype 3a derived SGR-Feo-S52 containing either the AII (T1056A, T1429I and S2204I) or SHI (P1220S, D1430H and S2204I) set of culture adaptive mutations (Saeed et al., 2012). These cells were incubated with the 4 compounds or DCV (AII: 5.2 ɳM and SHI 2.4 ɳM) for 48 h and harvested. Both genotype 3a SGRs were effectively inhibited by all 4 compounds (*p* < 0.05) (Fig. 6, Table 1). Additionally, we assayed the 4 compounds against genotype 1b by using Huh7.5 cells stably harboring the SGR-BM4-5 (Wyles et al., 2007). The 4 compounds were also able to significantly reduce HCV genotype 1b replication (*p* < 0.05) (Table 1). No significant differences were observed after treatment in replication with different genotypes (Fig. 6).

### 3.6 HCVcc infection is inhibited by Brazilian natural compounds

**Fig. 7:**
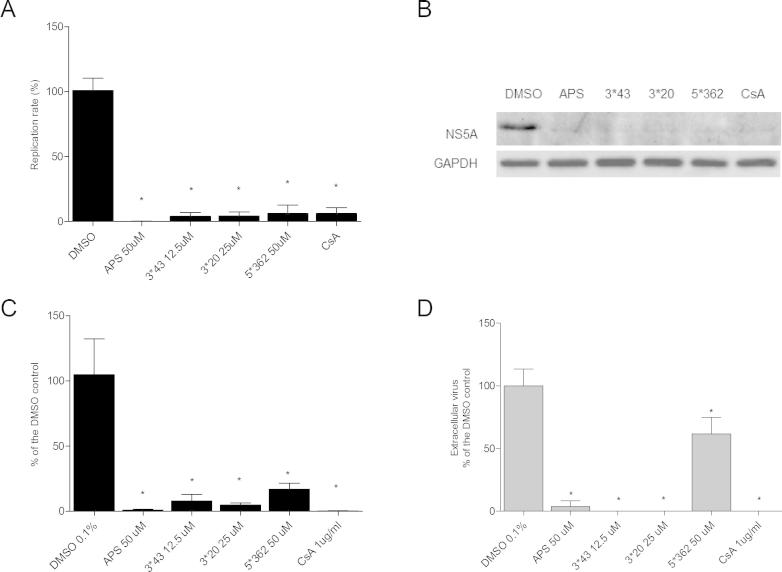
Effects of compounds APS, 3^∗^20, 3^∗^43 and 5^∗^362 on the HCV virus production. Huh7.5 cells were electroporated with Rluc-J6/JFH1 RNA, and 4 h later, specific concentrations of compounds were added. Replication efficiency was measured 48 h post-electroporation by measuring *Renilla* levels (A) and western blotting assays (B). Huh7.5 cells were infected with Rluc-J6/JFH1 virus and compounds were immediately added. Samples were harvested after 48 h and luminescence was measured (C). Cells were infected with JFH1 virus for 4 h, washed extensively to remove virus and treated with the compounds. After 48 h samples were harvested, and extracellular virus titrated (D). Mean values of three independent experiments each measured in triplicate including the standard deviation are shown. *P* < 0.05 was considered significant.

To determine the effect of the compounds APS, 3^∗^43, 3^∗^20 and 5^∗^362 on genome replication in the context of full length virus, we first used the Rluc-J6/JFH1 (FL-J6/JFH-5′C19Rluc2AUbi reporter) – a genotype 2a J6/JFH1 chimeric virus with *Renilla* luciferase fused to the HCV Core protein (Tscherne et al., 2006). Huh7.5 cells were electroporated with in vitro transcribed Rluc-J6/JFH1 RNA prior to incubation with the 4 compounds at 4 h. Replication was assessed by measuring *Renilla* luciferase levels at 48 h post-electroporation. Consistent with the SGR data, these compounds effectively blocked Rluc-J6/JFH1 replication (Fig. 7A). Protein expression levels were also significantly reduced in the presence of the compounds (Fig. 7B). APS was the most effective inhibitor of HCVcc replication, reducing replication by 500 fold at a concentration of 50 μM. CsA was included as a control for inhibition of genome replication.

To confirm that the compounds inhibited genome replication in the context of virus infection (as compared to RNA electroporation) we infected Huh7.5 cells with Rluc-J6/JFH1 HCVcc virus in the presence or absence of compounds for 48 h and again measured *Renilla* luciferase. As expected, HCVcc infection was significantly reduced in the presence of APS, 3^∗^43, 3^∗^20 and 5^∗^362 (Fig. 7C, Table 1).

We further confirmed the anti-HCV activity of the compounds by quantifying extracellular levels of virus after incubation of infected cells with the compounds. In this case Huh7.5 were infected with JFH1 virus for 4 h and subsequently treated for 48 h. Levels of released virus were significantly reduced by all 4 compounds (Fig. 7D, Table 1), although in this context 5^∗^362 had a less dramatic effect.

## 4 Discussion

HCV infection is a serious health problem and the new therapeutic regimes for the treatment of patients are very expensive and are associated with significant risk for the development of resistance. Therefore, the search for alternative therapies against HCV remains a valid aim, particularly in the context of low and middle-income countries that will not be able to afford the new drugs.

In this study, we screened a set of compounds extracted from Brazilian plants and we identified four compounds with potent inhibitory activity on HCV replication. These compounds are APS (EC_50_ = 2.3 μM), a natural alkaloid isolated from *M. ilicifolia*, the tetrahydrofuran lignans 3^∗^43 (EC_50_ = 4.0 μM) and 3^∗^20 (EC_50_ = 8.2 μM) and the secolignan 5^∗^362 (EC_50_ = 38.9 μM) from *P. blanda*. Our data demonstrated that HCV RNA and protein levels were dramatically reduced when the inhibitory effects of these compounds on HCV replication were analyzed using either subgenomic reporter SGR-Feo-JFH1 and the full-length Rluc-J6/JFH1.

The antiviral activity of alkaloids and lignans on HCV life cycle was previously described. Honokiol, a lignan isolated from leaves of *Magnolia officinais*, showed to have multiple effects on HCV infection, inhibiting entry, translation and replication in Huh7.5 cells using HCVcc, HCVpp, and subgenomic replicons (Lan et al., 2012). The reduction of protein and RNA levels was also shown by the treatment of cells in a subgenomic replicon system with 3-hydroxy caruilignan C (3-HCl-C) isolated from *Swietenia macrophylla* stems, which also increased the replication suppression when combined with IFN-α and protease or polymerase inhibitors (Wu et al., 2012). The flavonolignan Silymarin extracted from *Silybum marianum* (milk thistle) has shown recently to block virus entry, RNA and protein expression, virus production and cell to cell spread of virus (Wagoner et al., 2010). Additionally, this compound demonstrated a hepatoprotective effect on treated cells (Polyak et al., 2010). Myriberine A is an alkaloid isolated from *Myrioneuron faberi* and demonstrated inhibition against the HCV life cycle in vitro with a good therapeutic index (CC_50_/EC_50_) of greater than 12.0 in vitro for non-cytotoxic concentration (Huang et al., 2013). Oxymatrine and matrine are the two major alkaloid aqueous extracts from the *Sophora* root. Oxymatrine is reported to have antiviral activity against HCV in cell cultures and has shown hepatoprotective activity in an animal study (Chen et al., 2001; Liu et al., 1994). In a clinical perspective, the components Oxymatrine and matrine found in sophora roots have shown to reduce viral load and inhibition of liver fibrosis (Hussein et al., 2000; Kitazato et al., 2007). All these studies showed that natural lignans and alkaloids have potential for development as new bioactive molecules against HCV. Moreover, the extra effects of those compounds on HCV life cycle and clinical data demonstrated that further Brazilian compounds can present extra mode of action which need to be investigated.

Our results demonstrated that the compounds APS, 3^∗^43, 3^∗^20 and 5^∗^362 decreased HCV replication in a dose-dependent manner and acted to prevent RC formation. Using an Huh7.5 cell line stably harboring a subgenomic reporter we were able to demonstrate that treatment with compounds for 4 h did not inhibit RCs. In contrast, replication levels were reduced from 16 h of treatment when new RCs were formed, similar to transient assay performed with subgenomic reporter, suggesting that these compounds are acting on new RCs. In a previous study, Lyn et al. demonstrated that the treatment of Huh7.5 with lipid metabolism inhibitors disrupted the replication complexes by changing density and distribution of lipid droplets and consequently changing HCV RNA location which inhibited HCV replication (Lyn et al., 2009). However, the action of the compounds on pre-existing RCs was not clearly addressed.

In this context, reduction of HCV RNA and protein levels observed in our data could be consequence of the direct inhibition of viral enzymes (Ahmed-Belkacem et al., 2010; Bachmetov et al., 2012; Wagoner et al., 2010), the interference of these compounds with cellular factors involved in virus replication, or by inducing cellular antiviral effectors as has been shown previously (Gonzalez et al., 2009; Polyak et al., 2007; Rinck et al., 2001; Yi et al., 2011).

We were also able to show that the antiviral activity of Brazilian naturally occurring compounds was independent of HCV genotype and was not affected by variants described to confer resistance to Daclatasvir, a highly potent direct-acting antiviral drug targeting NS5A (Gao et al., 2010; Guedj et al., 2013; Lemm et al., 2010). Other plant-derived compounds have showed to be active on HCV life cycle independently of viral genotype or subtype (Choi et al., 2014; Haid et al., 2012), presenting an additional benefit to the current interferon-based HCV therapies or to the directly target antivirals which efficacy depend on viral genotypes. Haid et al. also demonstrated that viral resistance did not compromise the antiviral activity of a synthetic flavonoid-like compound against wild-type and mutant virus (Haid et al., 2012).

Moreover, most of the compounds did not affect HCV IRES driven translation indicating that the major antiviral mechanism is to directly inhibit virus genome replication. As an exception, the compound F8^∗^40, a natural kavalactone isolated from *Piper fuligineum*, showed significant but not dramatic effect on IRES-directed translation and corroborated with protein levels reduction in the presence of this compound. This data can suggest that the mode of action of this compound is related to the inhibition of IRES-mediated translation. The effect in baseline IRES translation was earlier showed by Gonzalez et al. by treating cells with the plant-derived flavonoid Quercetin which also had a strong inhibitory effect at 50 μM on HCV production in cell culture (Gonzalez et al., 2009).

In summary, our data demonstrate that natural alkaloids and lignans isolated from Brazilian plants dramatically inhibited HCV replication in vitro. Further analyses are in progress to elucidate other modes of action of those compounds. These data are the first description of Brazilian natural compounds possessing anti-HCV activity and as such may be useful in the development of future antiviral interventions for HCV and possibly other viral infections.

## Figures and Tables

**Table 1:** Inhibitory effect of Brazilian natural compounds on HCV replication.

				SGR-luc-JFH1 RC assay	SGR-JFH1 FEO RC assay	SGR-BM4-5 assay	SGR-Feo-S52 AII – SHI assay	J6/JFH1 HCVcc infection assay	JFH1 virus infection assay
Compound	EC_50_ (μM)	SI (CC_50_/EC_50_)	Concentration assays (μM)	% Inhibition					
APS	2.3	58.8	50	100	94	88	78–87	99	96
3^∗^43	4.0	4.7	12.5	92	88	75	96–98	92	100
3^∗^20	8.2	4.0	25	87	82	32	80–86	95	100
5^∗^362	38.9	1.9	50	68	52	62	88–88	83	38
CsA	NT	NT	1 μg/μL	93	95	94	100–100	100	100

EC_50_, effective concentration 50%; CC_50_, cytotoxic concentration 50%; SI, selective index; RC, Replication Complex; HCV, hepatitic C virus; NT, not tested; CsA, Cyclosporin A.

## References

[b0005] Ahmed-Belkacem A., Ahnou N., Barbotte L., Wychowski C., Pallier C., Brillet R., Pohl R.T., Pawlotsky J.M. (2010). Silibinin and related compounds are direct inhibitors of hepatitis C virus RNA-dependent RNA polymerase. Gastroenterology.

[b0010] Alter M.J. (2007). Epidemiology of hepatitis C virus infection. World J. Gastroenterol..

[b0015] Bachmetov L., Gal-Tanamy M., Shapira A., Vorobeychik M., Giterman-Galam T., Sathiyamoorthy P., Golan-Goldhirsh A., Benhar I., Tur-Kaspa R., Zemel R. (2012). Suppression of hepatitis C virus by the flavonoid quercetin is mediated by inhibition of NS3 protease activity. J. Viral Hepatol..

[b0020] Calland N., Dubuisson J., Rouillé Y., Séron K. (2012). Hepatitis C virus and natural compounds: a new antiviral approach?. Viruses.

[b0025] Chen Y., Li J., Zeng M., Lu L., Qu D., Mao Y., Fan Z., Hua J. (2001). The inhibitory effect of oxymatrine on hepatitis C virus in vitro. Zhonghua Gan Zang Bing Za Zhi.

[b0030] Chevaliez S., Pawlotsky J.M. (2007). Hepatitis C virus: virology, diagnosis and management of antiviral therapy. World J. Gastroenterol..

[b0035] Choi M., Kim Y.M., Lee S., Chin Y.W., Lee C. (2014). Mangosteen xanthones suppress hepatitis C virus genome replication. Virus Genes.

[b0040] Costa P.M., Ferreira P.M., Bolzani Vda S., Furlan M., de Freitas Formenton Macedo Dos Santos V.A., Corsino J., de Moraes M.O., Costa-Lotufo L.V., Montenegro R.C., Pessoa C. (2008). Antiproliferative activity of pristimerin isolated from *Maytenus ilicifolia* (Celastraceae) in human HL-60 cells. Toxicol. In Vitro.

[b0045] Dos Santos V.A., Leite K.M., da Costa Siqueira M., Regasini L.O., Martinez I., Nogueira C.T., Galuppo M.K., Stolf B.S., Pereira A.M., Cicarelli R.M., Furlan M., Graminha M.A. (2013). Antiprotozoal activity of quinonemethide triterpenes from *Maytenus ilicifolia* (Celastraceae). Molecules.

[b0050] Duarte M.C., Figueira G.M., Sartoratto A., Rehder V.L., Delarmelina C. (2005). Anti-Candida activity of Brazilian medicinal plants. J. Ethnopharmacol..

[b0055] Felippe L.G., Baldoqui D.C., Kato M.J., Bolzani Vda S., Guimaraes E.F., Cicarelli R.M., Furlan M. (2008). Trypanocidal tetrahydrofuran lignans from *Peperomia blanda*. Phytochemistry.

[b0060] Felippe L.G., Batista J.M., Baldoqui D.C., Nascimento I.R., Kato M.J., He Y., Nafie L.A., Furlan M. (2012). VCD to determine absolute configuration of natural product molecules: secolignans from *Peperomia blanda*. Org. Biomol. Chem..

[b0065] Fried M.W., Shiffman M.L., Reddy K.R., Smith C., Marinos G., Goncales F.L., Haussinger D., Diago M., Carosi G., Dhumeaux D., Craxi A., Lin A., Hoffman J., Yu J. (2002). Peginterferon alfa-2a plus ribavirin for chronic hepatitis C virus infection. N. Engl. J. Med..

[b0070] Gao M., Nettles R.E., Belema M., Snyder L.B., Nguyen V.N., Fridell R.A., Serrano-Wu M.H., Langley D.R., Sun J.H., O’Boyle D.R., Lemm J.A., Wang C., Knipe J.O., Chien C., Colonno R.J., Grasela D.M., Meanwell N.A., Hamann L.G. (2010). Chemical genetics strategy identifies an HCV NS5A inhibitor with a potent clinical effect. Nature.

[b0075] Gonzalez O., Fontanes V., Raychaudhuri S., Loo R., Loo J., Arumugaswami V., Sun R., Dasgupta A., French S.W. (2009). The heat shock protein inhibitor Quercetin attenuates hepatitis C virus production. Hepatology.

[b0080] Guedj J., Dahari H., Rong L., Sansone N.D., Nettles R.E., Cotler S.J., Layden T.J., Uprichard S.L., Perelson A.S. (2013). Modeling shows that the NS5A inhibitor daclatasvir has two modes of action and yields a shorter estimate of the hepatitis C virus half-life. Proc. Natl. Acad. Sci. USA.

[b0085] Gullo F.P., Sardi J.C., Santos V.A., Sangalli-Leite F., Pitangui N.S., Rossi S.A., de Paula E.S.A.C., Soares L.A., Silva J.F., Oliveira H.C., Furlan M., Silva D.H., Bolzani V.S., Mendes-Giannini M.J., Fusco-Almeida A.M. (2012). Antifungal activity of maytenin and pristimerin. Evid. Based Complement Alternat. Med..

[b0090] Hadziyannis S.J., Sette H., Morgan T.R., Balan V., Diago M., Marcellin P., Ramadori G., Bodenheimer H., Bernstein D., Rizzetto M., Zeuzem S., Pockros P.J., Lin A., Ackrill A.M. (2004). Peginterferon-alpha2a and ribavirin combination therapy in chronic hepatitis C: a randomized study of treatment duration and ribavirin dose. Ann. Intern. Med..

[b0095] Haid S., Novodomska A., Gentzsch J., Grethe C., Geuenich S., Bankwitz D., Chhatwal P., Jannack B., Hennebelle T., Bailleul F., Keppler O.T., Poenisch M., Bartenschlager R., Hernandez C., Lemasson M., Rosenberg A.R., Wong-Staal F., Davioud-Charvet E., Pietschmann T. (2012). A plant-derived flavonoid inhibits entry of all HCV genotypes into human hepatocytes. Gastroenterology.

[b0100] Huang S.D., Zhang Y., Cao M.M., Di Y.T., Tang G.H., Peng Z.G., Jiang J.D., He H.P., Hao X.J. (2013). Myriberine A, a new alkaloid with an unprecedented heteropentacyclic skeleton from *Myrioneuron faberi*. Org. Lett..

[b0105] Hussein G., Miyashiro H., Nakamura N., Hattori M., Kakiuchi N., Shimotohno K. (2000). Inhibitory effects of sudanese medicinal plant extracts on hepatitis C virus (HCV) protease. Phytother. Res..

[b0110] Kitazato K., Wang Y., Kobayashi N. (2007). Viral infectious disease and natural products with antiviral activity. Drug Discoveries Ther..

[b0115] Lan K.H., Wang Y.W., Lee W.P., Lan K.L., Tseng S.H., Hung L.R., Yen S.H., Lin H.C., Lee S.D. (2012). Multiple effects of Honokiol on the life cycle of hepatitis C virus. Liver Int..

[b0120] Lemm J.A., O’Boyle D., Liu M., Nower P.T., Colonno R., Deshpande M.S., Snyder L.B., Martin S.W., St Laurent D.R., Serrano-Wu M.H., Romine J.L., Meanwell N.A., Gao M. (2010). Identification of hepatitis C virus NS5A inhibitors. J. Virol..

[b0125] Liu J., Liu Y., Klaassen C.D. (1994). The effect of Chinese hepatoprotective medicines on experimental liver injury in mice. J. Ethnopharmacol..

[b0130] Lyn R.K., Kennedy D.C., Sagan S.M., Blais D.R., Rouleau Y., Pegoraro A.F., Xie X.S., Stolow A., Pezacki J.P. (2009). Direct imaging of the disruption of hepatitis C virus replication complexes by inhibitors of lipid metabolism. Virology.

[b0135] Macdonald A., Crowder K., Street A., McCormick C., Saksela K., Harris M. (2003). The hepatitis C virus non-structural NS5A protein inhibits activating protein-1 function by perturbing ras-ERK pathway signaling. J. Biol. Chem..

[b0140] Mann J. (2002). Natural products in cancer chemotherapy: past, present and future. Nat. Rev. Cancer.

[b0145] Manns M.P., McHutchison J.G., Gordon S.C., Rustgi V.K., Shiffman M., Reindollar R., Goodman Z.D., Koury K., Ling M., Albrecht J.K. (2001). Peginterferon alfa-2b plus ribavirin compared with interferon alfa-2b plus ribavirin for initial treatment of chronic hepatitis C: a randomised trial. Lancet.

[b0150] Pawlotsky J.M. (2014). New hepatitis C therapies: the toolbox, strategies, and challenges. Gastroenterology.

[b0155] Polyak S.J., Morishima C., Shuhart M.C., Wang C.C., Liu Y., Lee D.Y. (2007). Inhibition of T-cell inflammatory cytokines, hepatocyte NF-kappaB signaling, and HCV infection by standardized Silymarin. Gastroenterology.

[b0160] Polyak S.J., Morishima C., Lohmann V., Pal S., Lee D.Y., Liu Y., Graf T.N., Oberlies N.H. (2010). Identification of hepatoprotective flavonolignans from silymarin. Proc. Natl. Acad. Sci. USA.

[b0165] Rinck G., Birghan C., Harada T., Meyers G., Thiel H.J., Tautz N. (2001). A cellular J-domain protein modulates polyprotein processing and cytopathogenicity of a pestivirus. J. Virol..

[b0170] Ross-Thriepland D., Harris M. (2014). Insights into the complexity and functionality of hepatitis C virus NS5A phosphorylation. J. Virol..

[b0175] Saeed M., Scheel T.K., Gottwein J.M., Marukian S., Dustin L.B., Bukh J., Rice C.M. (2012). Efficient replication of genotype 3a and 4a hepatitis C virus replicons in human hepatoma cells. Antimicrob. Agents Chemother..

[b0180] Saito I., Miyamura T., Ohbayashi A., Harada H., Katayama T., Kikuchi S., Watanabe Y., Koi S., Onji M., Ohta Y. (1990). Hepatitis C virus infection is associated with the development of hepatocellular carcinoma. Proc. Natl. Acad. Sci. USA.

[b0185] Santos V.A., Regasini L.O., Nogueira C.R., Passerini G.D., Martinez I., Bolzani V.S., Graminha M.A., Cicarelli R.M., Furlan M. (2012). Antiprotozoal sesquiterpene pyridine alkaloids from *Maytenus ilicifolia*. J. Nat. Prod..

[b0190] Shepard C.W., Finelli L., Alter M.J. (2005). Global epidemiology of hepatitis C virus infection. Lancet Infect. Dis..

[b0195] Targett-Adams P., McLauchlan J. (2005). Development and characterization of a transient-replication assay for the genotype 2a hepatitis C virus subgenomic replicon. J. Gen. Virol..

[b0200] Tscherne D.M., Jones C.T., Evans M.J., Lindenbach B.D., McKeating J.A., Rice C.M. (2006). Time- and temperature-dependent activation of hepatitis C virus for low-pH-triggered entry. J. Virol..

[b0205] Wagoner J., Negash A., Kane O.J., Martinez L.E., Nahmias Y., Bourne N., Owen D.M., Grove J., Brimacombe C., McKeating J.A., Pecheur E.I., Graf T.N., Oberlies N.H., Lohmann V., Cao F., Tavis J.E., Polyak S.J. (2010). Multiple effects of silymarin on the hepatitis C virus lifecycle. Hepatology.

[b0210] Wakita T., Pietschmann T., Kato T., Date T., Miyamoto M., Zhao Z., Murthy K., Habermann A., Krausslich H.G., Mizokami M., Bartenschlager R., Liang T.J. (2005). Production of infectious hepatitis C virus in tissue culture from a cloned viral genome. Nat. Med..

[b0215] Wu S.F., Lin C.K., Chuang Y.S., Chang F.R., Tseng C.K., Wu Y.C., Lee J.C. (2012). Anti-hepatitis C virus activity of 3-hydroxy caruilignan C from *Swietenia macrophylla* stems. J. Viral Hepatol..

[b0220] Wyles D.L., Kaihara K.A., Vaida F., Schooley R.T. (2007). Synergy of small molecular inhibitors of hepatitis C virus replication directed at multiple viral targets. J. Virol..

[b0225] Wyles D.L., Kaihara K.A., Korba B.E., Schooley R.T., Beadle J.R., Hostetler K.Y. (2009). The octadecyloxyethyl ester of (S)-9-[3-hydroxy-2-(phosphonomethoxy) propyl]adenine is a potent and selective inhibitor of hepatitis C virus replication in genotype 1A, 1B, and 2A replicons. Antimicrob. Agents Chemother..

[b0230] Yi Z., Sperzel L., Nurnberger C., Bredenbeek P.J., Lubick K.J., Best S.M., Stoyanov C.T., Law L.M., Yuan Z., Rice C.M., MacDonald M.R. (2011). Identification and characterization of the host protein DNAJC14 as a broadly active flavivirus replication modulator. PLoS Pathog..

